# Analysis of the anxiety level and influencing factors during the coronavirus disease 2019 epidemic among the parents of students in China

**DOI:** 10.3389/fpubh.2023.1143836

**Published:** 2023-03-09

**Authors:** Jing Han, Xi Zhang, Shengchao Zhang, Yuting Li, Dongmei Zhang, Qingsong Chen

**Affiliations:** ^1^Shenzhen Bao'an District Central Hospital, Shenzhen, China; ^2^School of Public Health, Guangdong Pharmaceutical University, Guangzhou, China; ^3^Guangdong Provincial Engineering Research Center of Public Health Detection and Assessment, Guangdong Pharmaceutical University, Guangzhou, China

**Keywords:** parents, anxiety, GAD-7, coronavirus disease 2019, influencing factors

## Abstract

**Background:**

Due to the outbreak of Coronavirus Disease 2019, there has been a significant impact on the mental health of parents. However, no detailed study on the mental health status of parents has been conducted to date.

**Methods:**

This study was a cross-sectional used a whole-group random sampling method to conduct an online questionnaire survey with 102,883 parents in Guangdong Province, China, April 25, 2020 and May 14, 2020. Anxiety was assessed by using the Generalized Anxiety Disorder tool (GAD-7). Potential factors of anxiety were estimated using univariate analysis and multivariate logistic regression analysis by SPSS 22.0 statistical software.

**Results:**

Among the total 94,705 parents who have completed the questionnaire survey (92.05% response rate). The incidence of anxiety was 23.77%. Parents' anxiety symptoms are more likely to be caused by female family roles, higher levels of education, unemployed or jobless employment status, children not being an only child, and children having negative attitudes toward online courses.

**Conclusions:**

Our research shows that most parents experienced mild anxiety during the Coronavirus Disease 2019 epidemic. Our findings provide strong evidence for investigating and focusing on the mental health of this population during the COVID-19 epidemic. Therefore, governments and healthcare departments at all levels should actively provide psychological counseling services to relieve their anxiety symptoms.

## 1. Introduction

Since December 2019, COVID-19 – a cluster of acute respiratory illness with unknown causes (1) has spread rapidly in China and whole world, causing a serious damage to human life and health. The World Health Organization declared the coronavirus disease 2019 is a pandemic on March 11, 2020 (2), and the world entered a state of emergency. The COVID-19 not only brings people in China and other parts of the world the risk of death due to virus infection, but also brings unbearable psychological pressure (3, 4). In 2017, data from the Global Burden of Disease Study showed that the global prevalence of anxiety disorders was 3,721,764 cases per 100,000 people, ranking first among mental disorders (5). The sudden COVID-19 pandemic and the home isolation measures adopted by the government can be regarded as a kind of social isolation to some extent. It is understood that in the absence of interpersonal communication, anxiety disorders are more likely to occur and worsen (6). In an early study about investigating immediate psychological response during COVID-19 epidemic among general population in China reported that 53.8% of participants rated the psychological impact of the outbreak as moderate or severe (7). During COVID-19 pandemic, home isolation measures led to the physical and social isolation of parents, who suffered not only from work and financial problems, but also from the stresses of family life. Concerns about the health of family and friends and planning for the future in this new and uncertain situation. Especially with the delayed start of school for students nationwide and “stop classes without stopping” to carry out online classes for home learning, parents of students must supervise their children's learning, tutor homework and communicate with their teachers in a timely manner to ensure their children's normal learning status, in addition to protecting themselves and their families, resuming work and production pressure. All of these factors can lead to elevated levels of parental stress and accompanying symptoms of anxiety. Hence, a timely understanding of anxiety status is urgently needed.

At present, there is evidences that infectious diseases are associated with mental health problems in general population (8, 9), medical personnel (10, 11), patients, children and the elderly (12–14). However, no detailed study on the mental health status of parents of students has been conducted to date. Therefore, by understanding the anxiety level of Chinese students' parents during the COVID-19 epidemic and its influencing factors, our study is hereby carried out a cross-sectional study to provide a certain reference for relevant departments to carry out psychological intervention and guidance to the masses.

## 2. Materials and methods

### 2.1. Data collection

The anxiety level and related factors of the parents of students during the epidemic were evaluated by a cross-sectional survey. We used the online-based program “questionnaire star” as the survey vehicle and the education system as the backbone, the link to the questionnaire was sent to school heads in various cities in Guangdong Province. Participants voluntarily scans the QR code or clicks on the relevant link to fill in, and the survey respondent's informed consent is by default obtained. The questionnaire is equipped with mandatory questions and jump questions to ensure the completeness and logic of the answers. After the questionnaire is submitted, the researcher conducts manual screening, and deletes the answer sheets with obvious errors and “questionnaire response time ≤4 min” to ensure the authenticity and validity of the data obtained. The same IP address can only be answered once. The study was approved by the Medical Ethics Review Board of the School of Public Health, Guangdong Pharmaceutical University (IRB 2023–001), and complied with the Declaration of Helsinki guidelines. A total of 102,883 questionnaires from parents of students were collected anonymously from April 25, 2020 to May 14, 2020. 94705 questionnaires were available, the effective rate of the questionnaire was 92.05%.

### 2.2. Survey instrument

The content of the questionnaire includes general demographic characteristics and the Generalized Anxiety Disorder-7 (GAD-7). General information includes parental role, age, education level, employment status, per capita monthly household income, grade level of the child, access to internet classes, meals and exercise, etc. GAD-7 is an effective tool to identify cases of generalized anxiety disorder (15) and has good reliability and validity in previous studies on anxiety (16, 17). The Cronbach's alpha coefficient for the GAD-7 was 0.92. It has been recommended by the Psychiatric Branch of the Chinese Medical Association and the American Psychiatric Association. GAD-7 can be used to screen, diagnose and evaluate the severity of anxiety disorders, as well as social phobia, post–traumatic stress disorder and panic disorder (18). It takes less than 3 min to complete and easy to score (19). The GAD-7 measures the frequency of symptoms in 7 areas, according to the scoring criteria (20), including “not at all”, “a few days”, “more than a week” and “almost every day”, the corresponding scores are 0, 1, 2, and 3. The total score ranges from 0 to 21 points, the higher the total score, the higher the degree of anxiety disorder. Among them, a total score of 0 to 4 as no GAD, 5 to 9 as mild GAD, 10 to 14 as moderate GAD, and 15 to 21 as severe GAD. The Cronbach's alpha coefficient in the present study is 0.92.

### 2.3. Statistical analyses

Categorical variables were expressed as frequencies and percentages. Univariate analyses were conducted using the Kruskal–Wallis *H*-test. Independent correlations between anxiety symptoms and independent variables were assessed using multivariate logistic regression analysis. Model calibration was evaluated using the Hosmer-Lemeshow goodness-of-fit statistic. Statistical analyses were performed using SPSS 22.0 and a two-sided *P* < 0.05 was considered a statistically significant difference.

## 3. Results

### 3.1. Characteristics of the participants

Table 1 shows the characteristics of the participants. Of 94,705 parents, 22,514 parents experienced anxiety disorders during the outbreak. The majority of participants were between the ages of 36–45 years. The participants were mainly mothers 67,596 (71.38%), most parents had junior high school education or above 84,985 (89.74%), the majority of parents were employed 74,413 (78.57%), the per capita monthly household income was mainly below 3,000/month 34,532 (36.46%), nearly half of the children were in primary school 61,890 (65.35%) and not the majority of children are in primary school 61,890 (65.35%) and are not only children 81,452 (86.01%).

**Table 1:** General demographic characteristics of students' parents and their level of anxiety.

**Variable**	**Population (*n =* 94705)**	**No GAD (*n =* 72191)**	**Mild GAD (*n =* 19133)**	**Moderate GAD (*n =* 2354)**	**Severe GAD (*n =* 1027)**	***P***
**Family role**						<0.001
Father	22,520 (23.78)	18,017 (24.96)	3,751 (19.61)	467 (19.84)	285 (27.75)	
Mother	67,596 (71.38)	50,688 (70.21)	14,517 (75.87)	1,751 (74.38)	640 (62.32)	
Grandfather/ Maternal grandfather	734 (0.77)	595 (0.82)	107 (0.56)	22 (0.93)	10 (0.97)	
Grandmother/maternal grandmother	1,057 (1.12)	808 (1.12)	199 (1.04)	36 (1.53)	14 (1.36)	
Other	2,798 (2.95)	2,083 (2.89)	559 (2.92)	78 (3.32)	78 (7.60)	
**Age**						<0.001
≤25 years old	2,421 (2.56)	1,754 (2.43)	524 (2.74)	75 (3.19)	68 (6.62)	
26–35 years old	29,500 (31.15)	22,247 (30.82)	6,234 (32.58)	750 (31.86)	269 (26.19)	
36–45 years old	50,604 (53.43)	38,660 (53.55)	10,225 (53.44)	1,207 (51.27)	512 (49.85)	
46–55 years old	10,563 (11.15)	8,251 (11.43)	1,881 (9.83)	281 (11.94)	150 (14.61)	
≥56 years old	1617 (1.71)	1279 (1.77)	269 (1.41)	41 (1.74)	28 (2.73)	
**Degree of education**						<0.001
Primary school and below	9,720 (10.26)	7,142 (9.89)	2,077 (10.86)	342 (14.53)	159 (15.48)	
Junior middle school	46,373 (48.96)	35,244 (48.82)	9,491 (49.61)	1,152 (48.94)	486 (47.32)	
High school/secondary school	24,943 (26.34)	19,342 (26.79)	4,851 (25.35)	542 (23.02)	208 (20.25)	
College/undergraduate	13,312 (14.06)	10,183 (14.11)	2,657 (13.89)	307 (13.04)	165 (16.07)	
Graduate and above	357 (0.38)	280 (0.39)	57 (0.29)	11 (0.47)	9 (0.88)	
**Employment situation**						<0.001
In business	74,413 (78.57)	57,497 (79.65)	14,480 (75.68)	1,668 (70.86)	768 (74.78)	
Retirement	1,234 (1.31)	949 (1.31)	220 (1.15)	44 (1.87)	21 (2.05)	
Unemployed	19,058 (20.12)	13,745 (19.04)	4,433 (23.17)	642 (27.27)	238 (23.17)	
**Household per capita income**						<0.001
Less 3000 Yuan/month	34,532 (36.46)	25,464 (35.27)	7505 (39.23)	1119 (47.54)	444 (43.23)	
3000–4999 Yuan/month	31,396 (33.15)	24,158 (33.46)	6,232 (32.57)	692 (29.40)	314 (30.57)	
5000–7999 Yuan/month	16,391 (17.31)	12,841 (17.79)	3,102 (16.21)	299 (12.69)	149 (14.51)	
8000–9999 Yuan/month	5,863 (6.19)	4,567 (6.33)	1,130 (5.91)	115 (4.89)	51 (4.97)	
10000–20000 Yuan/month	4,964 (5.24)	3,921 (5.43)	901 (4.71)	100 (4.25)	42 (4.09)	
More than 20000 Yuan/month	1,559 (1.65)	1,240 (1.72)	263 (1.37)	29 (1.23)	27 (2.63)	
**Children's grade**						<0.001
Kindergarten	5,040 (5.32)	3,857 (5.34)	976 (5.11)	133 (5.65)	74 (7.21)	
Primary school	61,890 (65.35)	46,672 (64.65)	12,864 (67.23)	1,652 (70.18)	702 (68.36)	
Junior middle school	18,085 (19.10)	14,041 (19.45)	3,504 (18.31)	381 (16.19)	159 (15.48)	
High school	7,160 (7.56)	5,627 (7.79)	1,332 (6.96)	149 (6.33)	52 (5.06)	
University	2,530 (2.67)	1,994 (2.77)	457 (2.39)	39 (1.65)	40 (3.89)	
**Is the child the only**						<0.001
Yes	13,253 (13.99)	10,411 (14.42)	2,408 (12.59)	297 (12.62	137 (13.34)	
No	81,452 (86.01)	61,780 (85.58)	16,725 (87.41)	2,057 (87.38)	890 (86.66)	

### 3.2. Univariate analysis of potential factors related to symptoms of anxiety among parents during the COVID-19 epidemic

The overall prevalence of anxiety symptoms was 23.77%. Of these, 72,191 (76.23%) had no anxiety symptoms (0–4); 19,133 (20.21%) had mild anxiety symptoms (5–9); 23,54 (2.49%) had moderate anxiety symptoms (10–14); and 1,027 (1.07%) had severe anxiety symptoms (15–21). Table 1 shows that family role, age, education, employment status, monthly income per capita, grade level of the child, and whether or not the child is an only child are all potential factors related to anxiety symptoms (*P* < 0.001).

#### 3.2.1. Relationship between family and self COVID-19 infection status and anxiety symptoms

As the survey was conducted during a period of home isolation for outbreak prevention and control, it was particularly important to understand the impact of parents' own and their families' physical health on anxiety. Table 2 presents that participants who are in good health and have no history of exposure are less likely to experience anxiety (76.56% had no anxiety symptoms). The level of anxiety was higher in cases with confirmed patients than in other cases (*P* < 0.001).

**Table 2:** The influence of family members and their own COVID-19 infection on anxiety.

**Variable**	**NoGAD**	**Mild GAD**	**Moderate GAD**	**Severe GAD**	***P***
**In good health and no contact history**					<0.001
Yes	70,745 (98.00)	18,491 (96.64)	2,228 (94.65)	938 (91.33)	
No	1,446 (2.00)	642 (3.36)	126 (5.35)	89 (8.67)	
**Suspected patients**					<0.001
Yes	88 (0.12)	56 (0.29)	22 (0.93)	14 (1.36)	
No	72,103 (99.88)	19,077 (99.71)	2,332 (99.07)	1,013 (98.64)	
**Confirmed patients**					<0.001
Yes	66 (0.09)	54 (0.28)	20 (0.85)	10 (0.97)	
No	72,125 (99.91)	19,079 (99.72)	2,334 (99.15)	1,017 (99.03)	
**Asymptomatic infection**					<0.001
Yes	1,767 (2.45)	655 (3.42)	122 (5.18)	49 (4.77)	
No	70,424 (97.55)	18,478 (96.58)	2,232 (94.82)	978 (95.23)	
**Close contact**					<0.001
Yes	167 (0.23)	78 (0.41)	19 (0.81)	5 (0.49)	
No	72,024 (99.77)	19,055 (99.59)	2,335 (99.19)	1,022 (99.51)	

#### 3.2.2. Relationship between children's access to internet classes, meals, exercise, and anxiety symptoms

Both children's and parents' attitudes toward Internet classes were influential factors in anxiety symptoms. Those with “strongly dislike” (0.53% had no anxiety symptoms) and “doesn't matter” (11.22% had no anxiety symptoms) attitudes had higher levels of anxiety than the rest of the group (Table 3).

**Table 3:** The impact of children's online classes on the anxiety of parents.

**Variable**	**No GAD**	**Mild GAD**	**Moderate GAD**	**Severe GAD**	***P***
**Whether online courses**					0.089
Yes	65,835 (91.20)	17,408 (90.98)	2,129 (90.44)	919 (89.48)	
No	6,356 (8.80)	1,725 (9.02)	225 (9.56)	108 (10.52)	
**Children's attitudes**					<0.001
Adore	4,691 (7.13)	819 (4.70)	123 (5.78)	78 (8.49)	
Liked	20,047 (30.45)	4,118 (23.66)	400 (18.79)	156 (16.97)	
Fair	39,487 (59.97)	11,724 (67.35)	1,457 (68.44)	598 (65.07)	
Dislike	1,263 (1.92)	593 (3.41)	99 (4.65)	48 (5.22)	
Disgust	347 (0.53)	154 (0.88)	50 (2.34)	39 (4.25)	
**Parents' attitudes**					<0.001
Agree	44,756 (67.98)	10,440 (59.97)	1,131 (53.12)	520 (56.58)	
Disagree	13,692 (20.80)	4,822 (27.70)	733 (34.43)	252 (27.42)	
Doesn't matter	7,387 (11.22)	2,146 (12.33)	265 (12.45)	147 (16.00)	

The frequency of breakfast “6–7 times/week” (70.79% had no anxiety symptoms) and the option “no significant change” (75.46%, 62.11%, 66.21%, 70.35%, 57.53% had no anxiety symptoms) had the lowest number of anxieties, while the options “never eat breakfast” (2.32% had no anxiety symptoms) and “no intake” (2.12%,1.81%,2.04%,4.37%,6.30% had no anxiety symptoms) were both more anxious than the other cases (Table 4).

**Table 4:** The impact of a children's dietary situation on anxiety symptoms of parents.

**Variable**	**No GAD**	**Mild GAD**	**Moderate GAD**	**Severe GAD**	***P***
**Breakfast frequency**					<0.001
Never	1,674 (2.32)	506 (2.64)	103 (4.38)	77 (7.50)	
1–2 times/week	5,907 (8.18)	1,905 (9.96)	280 (11.89)	145 (14.12)	
3–5 times/week	13,504 (18.71)	4,195 (21.93)	540 (22.94)	226 (22.00)	
6–7 times/week	51,106 (70.79)	12,527 (65.47)	1,431 (60.79)	579 (56.38)	
**Staple food**					<0.001
No intake	1,534 (2.12)	501 (2.62)	84 (3.57)	39 (3.80)	
Increase	13,348 (18.49)	4,387 (22.93)	563 (23.92)	130 (12.66)	
Decrease	2,836 (3.93)	1,393 (7.28)	307 (13.04)	68 (6.62)	
No visible change	54,473 (75.46)	12,852 (67.17)	1,400 (59.47)	790 (76.92)	
**Vegetables and fruits**					<0.001
No intake	1,314 (1.82)	374 (1.95)	69 (2.93)	45 (4.38)	
Increase	22,747 (31.51)	6,881 (35.96)	877 (37.26)	190 (18.50)	
Decrease	3,289 (4.56)	1,624 (8.49)	347 (14.74)	71 (6.91)	
No visible change	44,841 (62.11)	10,254 (53.60)	1,061 (45.07)	721 (70.21)	
**Meat, eggs and fishery**					<0.001
No intake	1,475 (2.04)	377 (1.97)	72 (3.06)	33 (3.21)	
Increase	17,865 (24.75)	5,998 (31.35)	791 (33.60)	165 (16.07)	
Decrease	5,053 (7.00)	1,996 (10.43)	360 (15.29)	88 (8.57)	
No visible change	47,798 (66.21)	10,762 (56.25)	1,131 (48.05)	741 (72.15)	
**Soy products**					<0.001
No intake	3,154 (4.37)	864 (4.52)	134 (5.69)	47 (4.58)	
Increase	11,081 (15.35)	3,704 (19.36)	485 (20.60)	111 (10.81)	
Decrease	7,171 (9.93)	2,840 (14.84)	474 (20.14)	100 (9.74)	
No visible change	50,785 (70.35)	11,725 (61.28)	1,261 (53.57)	769 (74.87)	
**Dairy products**					<0.001
No intake	4,550 (6.30)	1,129 (5.90)	203 (8.62)	60 (5.84)	
Increase	18,120 (25.10)	5,672 (29.65)	709 (30.12)	155 (15.09)	
Decrease	7,992 (11.07)	2,972 (15.53)	509 (21.62)	108 (10.52)	
No visible change	41,529 (57.53)	9,360 (48.92)	933 (39.64)	704 (68.55)	

In terms of the children exercise profile, big balls (football, basketball, etc.), taekwondo, skipping rope, brisk walking/jogging, and whether or not the exercise was regular were all potential factors on anxiety symptoms, while running, dancing, and practicing musical instruments were not potential factors in anxiety symptoms. Choosing Taekwondo (1.68% had no anxiety symptoms) as a form of exercise is associated with higher levels of anxiety than other sports (Table 5).

**Table 5:** The influence of children's sports status on parents' anxiety.

**Variable**	**No GAD**	**Mild GAD**	**Moderate GAD**	**Severe GAD**	***P***
**Running**					0.624
Yes	26,826 (37.16)	7,186 (37.56)	904 (38.40)	329 (32.04)	
No	45,365 (62.84)	11,947 (62.44)	1,450 (61.60)	698 (67.96)	
**Big balls (football, basketball, etc)**					0.004
Yes	11,719 (16.23)	3,274 (17.11)	377 (16.02)	190 (18.50)	
No	60,472 (83.77)	15,859 (82.89)	1,977 (83.98)	837 (81.50)	
**Taekwondo**					<0.001
Yes	1,214 (1.68)	393 (2.05)	78 (3.31)	54 (5.26)	
No	70,977 (98.32)	18,740 (97.95)	2,276 (96.69)	973 (94.74)	
**Dancing**					0.885
Yes	11,076 (15.34)	2,942 (15.38)	364 (15.46)	157 (15.29)	
No	61,115 (84.66)	16,191 (84.62)	1,990 (84.54)	870 (84.71)	
**Jumping rope**					0.016
Yes	32,215 (44.62)	8,573 (44.81)	985 (41.84)	337 (32.81)	
No	39,976 (55.38)	10,560 (55.19)	1,369 (58.16)	690 (67.19)	
**Brisk walking/jogging**					0.031
Yes	29,511 (40.88)	7,806 (40.80)	914 (38.83)	337 (32.81)	
No	42,680 (59.12)	11,327 (59.20)	1,440 (61.17)	690 (67.19)	
**Musical instrument practice**					0.593
Yes	4,794 (6.64)	1,228 (6.42)	170 (7.22)	104 (10.13)	
No	67,397 (93.36)	17,905 (93.58)	2,184 (92.78)	923 (89.87)	
**Whether regular exercise**					0.000
Yes	35,528 (49.21)	8,918 (46.61)	1,037 (44.05)	503 (48.98)	
No	36,663 (50.79)	10 ,215 (53.39)	1,317 (55.95)	524 (51.02)	

### 3.3. Multivariate logistic regression analysis of factors significantly associated with anxiety symptoms among parents during the COVID-19 epidemic

In multiple logistic regression analysis, mother (OR = 1.265, 95% CI: 1.215–1.318) and (maternal) grandmother (OR = 1.323, 95% CI: 1.100–1.592), college/bachelor's degree (OR = 1.098, 95% CI: 1.024–1.177), unemployed or jobless (OR = 1.213, 95% CI: 1.166–1.263), child not an only child (OR = 1.070, 95% CI: 1.020–1.122), child's attitude toward online classes was fair (OR = 1.534, 95% CI: 1.429–1.647), disgusted (OR = 2.485, 95% CI: 2.217–2.786), very averse (OR = 2.906, 95% CI: 2.430–3.476) were more likely to show symptoms of anxiety.

Age: 26–35 years (OR = 0.874, 95% CI: 0.765–0.998), 36–45 years (OR = 0.853, 95% CI: 0.748–0.973), 46–55 years (OR = 0.777, 95% CI: 0.677–0.891), ≥56 years (OR = 0.675, 95% CI. 0.546–0.834), lower secondary education (OR = 0.935, 95% CI: 0.885–0.988), per capita monthly income: 3000–4999/month (OR = 0.870, 95% CI: 0.837–0.904), 5000–7999/month (OR = 0.818, 95% CI: 0.779- 0.858), 8000–9999 /month (OR = 0.841, 95% CI: 0.783–0.903), 10,000–20,000 /month (OR = 0.805, 95% CI: 0.744–0.871), 20,000 or more/month (OR = 0.740, 95% CI: 0.646–0.848), children's breakfast frequency 6–7 times/week (OR = 0.810,95%CI: 0.732–0.897) had a significant protective effect against parents developing symptoms of anxiety. See in Table 6.

**Table 6:** The logistic regression analysis of the influencing factors of parents.

**Variable**	***P***	**OR (95%CI)**
**Family role**		
Father	-	1.000
Mother	<0.001	1.265 (1.215–1.318)
Grandfather/maternal grandfather	0.904	0.985 (0.774–1.254)
Grandmother/maternal grandmother	0.003	1.323 (1.100–1.592)
Other	0.236	1.077 (0.953–1.218)
**Age**		
≤25 years old	-	1.000
26–35 years old	0.047	0.874 (0.765–0.998)
36–45 years old	0.018	0.853 (0.748–0.973)
46–55 years old	<0.001	0.777 (0.677–0.891)
≥56 years old	<0.001	0.675 (0.546–0.834)
**Degree of education**		
Primary school and below	-	1.000
Junior middle school	0.016	0.935 (0.885–0.988)
High school/secondary school	0.095	0.950 (0.894–1.009)
College/undergraduate	0.008	1.098 (1.024–1.177)
Graduate and above	0.953	0.992 (0.750–1.311)
**Employment situation**		
In business	-	1.000
Retirement	0.076	1.157 (0.985–1.358)
Unemployed	<0.001	1.213 (1.166–1.263)
**Household per capita income**		
Less 3000 Yuan/month	-	1.000
3000–4999 Yuan/month	<0.001	0.870 (0.837–0.904)
5000–7999 Yuan/month	<0.001	0.818 (0.779–0.858)
8000–9999 Yuan/month	<0.001	0.841 (0.783–0.903)
10000–20000 Yuan/month	<0.001	0.805 (0.744–0.871)
More than 20000 Yuan/month	<0.001	0.740 (0.646–0.848)
**Children's grade**		
Kindergarten	-	1.000
Primary school	0.144	1.058 (0.981–1.141)
Junior middle school	0.604	1.022 (0.941–1.110)
High school	0.628	0.977 (0.890–1.073)
University	0.519	0.960 (0.850–1.086)
**Is the child the only**		
Yes	-	1.000
No	0.006	1.070 (1.020–1.122)
**Children's attitudes**		
Adore	-	1.000
Liked	0.101	1.065 (0.988–1.148)
Fair	<0.001	1.534 (1.429–1.647)
Dislike	<0.001	2.485 (2.217–2.786)
Disgust	<0.001	2.906 (2.430–3.476)
**Breakfast frequency**		
Never	-	1.000
1–2 times/week	0.576	1.032 (0.923–1.155)
3–5 times/week	0.886	0.992 (0.893–1.103)
6–7 times/week	<0.001	0.810 (0.732–0.897)

## 4. Discussion

During COVID-19 pandemic, there was widespread concern about the mental health status of health care workers, children, older people and so on. There is little research on parental mental health issues. Parents, as the backbone of society and families, are under more pressure than usual. On the one hand, some families are under increased financial pressure due to economic changes and rising unemployment. On the other hand, home isolation measures has led to parents working at home while caring for and educating their children, and the double pressure has brought about a surge in anxiety among parents. Our study shows that the number of people with anxiety accounted for 23.77% of the total number of people. The mood of anxious people is mainly mild anxiety, which is similar to the survey results of Sun et al. (21). Parents with multiple children are more likely to be anxious and have higher levels of anxiety. This phenomenon is likely due to parents have more to worry about as they juggle their daily work and life while tutoring or supervising multiple children online. For students with negative attitudes toward online classes, the level of parental anxiety is even higher. Students usually receive information passively and lack active communication in online classes, and over time they may lose interest in online courses (22, 23). Parents are generally very concerned about their children's academic performance, and their children's negative attitudes toward online classes can add to their concerns.

In terms of meals, the more frequently children eat breakfast, the lower the anxiety of parents. This may be due to the fact that both parents and children are confined to their home during home isolation, and the prolonged periods of confinement make people prone to emotional instability. The regularity of children's eating habits will ease the irritability of parents. Isolation at home enables parents to supervise and feed their children, showing healthier eating habits (24). Our study found children's intake of all types of food did not change much compared to usual during the epidemic. These findings were consistent with a cross-sectional study in the China, which showed that 65% of participants did not change their eating habits during the epidemic (25) This study was conducted during the recovery period of the epidemic when people's lives were gradually returning to normal compared to the outbreak period, and dietary patterns were likely to be consistent with usual.

In addition to the influencing factors brought by children, parents' own conditions can also cause anxiety. In the family, mothers and grandmothers have relatively high levels of anxiety. It was said to be due to a heightened sensitivity of females to the threat (26), the result has implications for the identification of risk groups and interventions. Also anxiety may be related to estrogen levels, with studies showing a significant increase in anxiety levels during the first menstrual period and in post-menopausal women (27) It has been shown that circulating estradiol increases abruptly from prepubertal to adult levels during the sexual developmental phase, and therefore the risk of anxiety symptoms was significantly increased at the first menstrual period (28, 29). Among the influencing factors of age, parents of young students are more likely to have anxiety, which is similar to previous studies on age and anxiety (29–31). Older parents will have some financial means (some savings) even in the face of the epidemic, while younger parents will generally have less financial means than older ones (32). On the other hand, young people rely more on getting news about the epidemic from the Internet, and they receive more negative news than parents of older students. This may lead to anxiety about their future and concerns about their family and children. At this time, parents of students should maintain an objective and rational attitude in the face of numerous reports, and adjust their personal mentality.

There are signs that the increase in the number of patients and suspected cases, as well as the increase in the number of provinces and countries affected by the epidemic, has triggered public concerns about being infected in this epidemic. (33). The results of present study show that parents' anxiety about the epidemic is related to whether they and their family members are infected with the new crown pneumonia virus.

Research by Hinz et al. (34) also suggest that anxiety is related to unemployment and low income. As expected, the unemployed parents are more likely to have anxiety (35). The lower the income level, the higher their anxiety. Studies have shown that low-income households are more likely to experience financial hardship (e.g. loss of employment or pay cuts) (36). Income sources are suspended, and prices of epidemic prevention materials and daily necessities have risen during the epidemic. Income and expenditure are a huge challenge. Parents with relatively high incomes and stable employment have sufficient savings and resources to cope with the economic crisis brought about by the epidemic, while parents with low monthly household incomes will have more worries about their own and their families' future. On the other hand, the negative emotions of low-income parents may also affect their children, causing children from low-income families to show some degree of anxiety (37). These results suggest that financial support and psychological support are effective ways to alleviate anxiety during the epidemic.

At this particular time, anxiety is just as worrying as the virus. It is even more important for parents to adapt themselves and their children physically and mentally to protect themselves from the epidemic. There are several suggestions for this: First, relax and be optimistic. When you feel down, you can talk with your family and exercise together to relieve anxiety. Second, keep a regular schedule and healthy. During home isolation, it is also necessary to maintain regular work and rest, set an example, cultivate children's good living habits, and create a healthy and positive family atmosphere, which is more conducive to the removal of anxiety. Thirdly, the relevant authorities can help people through this difficult time by providing rent relief, reducing taxes and fees, stabilizing employment and granting temporary subsidies.

The limitations of this study is that the sample comes from Guangdong Province, but the distribution is uneven, and it cannot yet better represent the national situation; In addition, the collection of questionnaire is obtained through the student group contacting parentsand the results only represent a part of the population. In the future, researchers will expand representation of population and increase the sample size in all cities across the country, if possible, further to carry out cohort studies, and conduct research on the anxiety intervention of Chinese residents.

## 5. Conclusions

This study analyzed the anxiety level and related factors of the parents of students during the epidemic, and found that 23.77% of the parents had anxiety symptoms. Although the degree of anxiety of parents is mild, but the long-term lack of interpersonal communication is very likely to increase the degree of anxiety. Therefore, during the period of coronavirus disease 2019, governments and healthcare departments at all levels should actively provide psychological counseling services to relieve their anxiety symptoms.

## Data availability statement

The raw data supporting the conclusions of this article will be made available by the authors, without undue reservation.

## Ethics statement

The studies involving human participants were reviewed and approved by the Medical Ethics Review Board of the School of Public Health, Guangdong Pharmaceutical University. Written informed consent for participation was not required for this study in accordance with the national legislation and the institutional requirements.

## Author contributions

XZ, JH, YL, and DZ were involved in setting up the study and data collection. SZ, YL, and QC contributed to the data analysis. XZ drafted the manuscript. All authors approved the final version of the paper.
